# Exploring the Microbiome in Breast Cancer: The Role of *Fusobacterium nucleatum* as an Onco-Immune Modulator

**DOI:** 10.3390/microorganisms13091995

**Published:** 2025-08-27

**Authors:** Alessandra D’Angelo, Anna Zenoniani, Martina Masci, Gitana Maria Aceto, Adriano Piattelli, Maria Cristina Curia

**Affiliations:** 1 Department of Medical, Oral and Biotechnological Sciences, “G. d’Annunzio” University of Chieti-Pescara, Via dei Vestini, 66100 Chieti, Italy; alessandra.dangelo@unich.it (A.D.); anna.zenoniani@studenti.unich.it (A.Z.); martina.masci002@studenti.unich.it (M.M.); 2 Department of Science, “G. d’Annunzio” University of Chieti-Pescara, Via dei Vestini, 66100 Chieti, Italy; gitana.aceto@unich.it; 3 School of Dentistry, Saint Camillus International University of Health and Medical Sciences (UniCamillus), 00131 Rome, Italy; apiattelli51@gmail.com; 4 Facultad de Medicina, UCAM Universidad Católica San Antonio de Murcia, 30107 Murcia, Spain

**Keywords:** microbiome, breast cancer, breast milk, *Fusobacterium nucleatum*, Wnt/β-catenin, immune response, Fap2, FadA, TIGIT, estrobolome, MDSC

## Abstract

The breast microbiome remains stable throughout a woman’s life. The breast is not a sterile organ, and its microbiota exhibits a distinct composition compared to other body sites. The breast microbiome is a community characterized by an abundance of *Proteobacteria* and *Firmicutes*, which represent the result of host microbial adaptation to the fatty acid environment in the tissue. The breast microbiome demonstrates dynamic adaptability during lactation, responding to maternal physiological changes and infant interactions. This microbial plasticity modulates local immune responses, maintains epithelial integrity, and supports tissue homeostasis, thereby influencing both breast health and milk composition. Disruptions in this balance, the dysbiosis, are closely linked to inflammatory breast conditions such as mastitis. Risk factors for breast cancer (BC) include genetic mutations, late menopause, obesity, estrogen metabolism, and alterations in gut microbial diversity. Gut microbiota can increase estrogen bioavailability by deconjugating estrogen-glucuronide moieties. Perturbations of this set of bacterial genes and metabolites, called the estrobolome, increases circulating estrogens and the risk of BC. *Fusobacterium nucleatum* has recently been associated with BC. It moves from the oral cavity to other body sites hematogenously. This review deals with the characteristics of the breast microbiome, with a focus on *F. nucleatum*, highlighting its dual role in promoting tumor growth and modulating immune responses. *F. nucleatum* acts both on the Wnt/β-catenin pathway by positively regulating MYC expression and on apoptosis by inhibiting caspase 8. Furthermore, *F. nucleatum* binds to TIGIT and CEACAM1, inhibiting T-cell cytotoxic activity and protecting tumor cells from immune cell attack. *F. nucleatum* also inhibits T-cell function through the recruitment of myeloid suppressor cells (MDSCs). These cells express PD-L1, which further reduces T-cell activation. A deeper understanding of *F. nucleatum* biology and its interactions with host cells and co-existing symbiotic microbiota could aid in the development of personalized anticancer therapy.

## 1. Introduction

Breast cancer (BC) is the second cause of death related to cancer in the women worldwide [1]. It is clinically heterogeneous, with a variety of subtypes identified according to the molecular nature. Although other clinical parameters also have an impact, the microbiota appears to be an increasingly emerging risk factor. Intratumoral bacteria present in BC, although low in biomass, can promote metastatic colonization without affecting primary tumor growth [2]. It was shown that the gut microbiota influences the onset and development of BC through different processes; these mechanisms can be represented by the control of the immunity activity, the modification of estrogen levels, and the production of bacterial metabolites, which may affect cancer cells themselves and their habitat [3]. The progression of BC is driven by an increase in inflammatory mediators, generated by disruption of host and microbiota balance, leading to dysbiosis [4,5]. Antibiotic-induced dysbiosis has been shown to cause a significant increase in myeloid cells that express high levels of arginase-1 and IL-6, known as suppressive/inflammatory molecules, at the cancer site [6].

The intracellular tumor microbiota is a component of tumor tissue that has been documented in several types of cancer. However, its biological functions remain unclear. A potential link between the breast microbiota and maternal health, infant development, and the pathogenesis of breast cancer has been hypothesized, although this remains an emerging and underexplored area of research [7,8]. Omics studies could clarify the roles of normal and intratumoral spread of microbiota and how they interact with the host’s physiological or clinical conditions. This could lead to advances in personalized cancer treatment [9,10].

The breast is not a sterile organ, and its microbiota exhibits a distinct composition compared to other body sites [11,12,13]. Unlike other microbiomes, the breast microbiome remains stable across various factors, including pregnancy history (previous births vs. nulliparous women), age of the individual, presence or absence of breast malignancy, geographic location and ethnicity, sample collection site within the breast, and sequencing technologies used for analysis [11]. Both the milk and tissue of the breast contain a diverse bacterial community that has an impact on the healthy development of children’s intestines and on maintaining women’s health. The diversity of breast microbiota is comparable to that of the gut microbiome but significantly higher than that observed in the vaginal microbiome [14].

**Figure 1:**
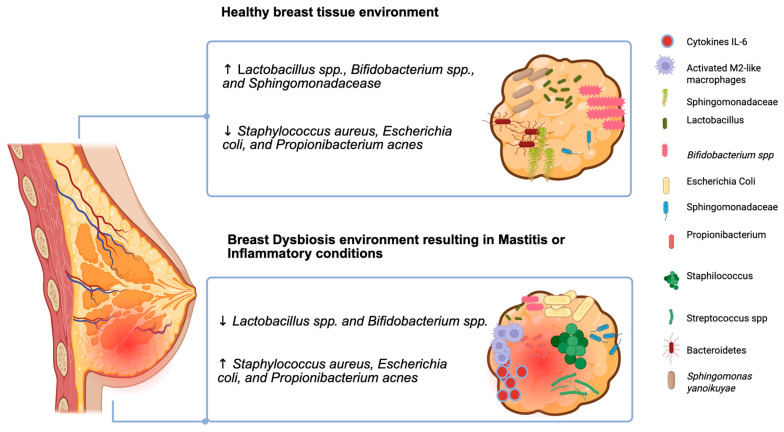
**Healthy breast versus inflammatory condition.** The breast microbiome in health is characterized by a balanced microbial community dominated by commensals such as *Lactobacillus*, *Bifidobacterium*, and *Sphingomonas*, supporting immune tolerance and neonatal gut colonization. In contrast, dysbiosis during inflammatory conditions such as mastitis or maternal obesity leads to an overgrowth of opportunistic pathogens (e.g., *Staphylococcus aureus* and *E. coli*), immune cell infiltration, and altered cytokine profiles. These changes can potentially disrupt lactation and compromise the long-term health of both infant and mothers [15]. Image created with BioRender (https://biorender.com; last accessed on 24 August 2025).

Determined bacteria found in breast tissue were also detected in other sites of the body like *Lactobacillus iners* and *Prevotella* (vagina), *Enterobacteriaceae* (gastrointestinal tract), *Fusobacterium* and *Streptococcus* (oral cavity), *Propionibacterium* and *Micrococcus* (skin), and *Pseudomonas* (respiratory tract). Both species with healthy properties such as *Lactobacillus* and *Bifidobacterium* and known pathogenic species like *Enterobacteriaceae*, *Pseudomonas* and *Streptococcus agalactiae* have been found [11]. The human breast harbors a unique and diverse microbiome that is distinct from microbial communities found in other body sites, including the gut, skin, and vagina [12]. For the first time in 2014 in two autonomy research studies, the presence of a native breast microbiome was detected [11,12], evidence that the breast hosts a large bacterial community [12]. Since the breast consists mainly of adipose tissue with a large vascularization and lymphatic drainage, it is a supportive habitat for bacterial expansion, in particular *Proteobacteria* and *Firmicutes* [13] (Figure 1).

Bacterial species such as *Bacillus* sp., *Enterobacteriaceae* sp., and *Staphylococcus* sp. were detected in breast tissue by bacterial cultivation, which highlighted living bacterial species [12]. Other bacterial species were also identified after tissue stratification and regulation for known confounding factors and factors that may affect the microbiome (age of patient, race, and hospital) [16,17]. The healthy controls and high-risk benign tissue samples show a comparable composition of microbiome, represented by great mean relative abundance of 11 genera: *Propionibacterium*, *Finegoldia*, *Granulicatella*, *Streptococcus*, *Anaerococcus*, *Ruminococcaceae*, *Corynebacterium 1*, *Alicyclobacillus*, *Odoribacter*, *Lactococcus*, and *Esherichica/Shigella* [18]. Recent research suggests that a portion of the mammary microbiome may originate from microbial translocation pathways involving both the gastrointestinal tract and the skin. These microbes can reach the mammary gland via systemic circulation, the nipple–areolar complex (including its ductal openings), or through direct oral contact during breastfeeding or sexual activity. This multifaceted transmission underscores the dynamic and interconnected nature of maternal microbial ecosystems [9]. One of the possible hypotheses of colonization could be explained by the movement from other body sites, such as the intestine or the oral cavity [19]. The microbiome could also play a defensive function towards the breast because the immune cells stimulate healthy tissue and the degradation of carcinogenic metabolites could be carried out by the activity of resident bacteria, always in a defensive view and maintenance of healthy tissue [11]. *Fusobacterium nucleatum* (*F. nucleatum*) is among the bacteria that colonize the breast. It is an obligate anaerobic Gram-negative bacterium belonging to the genus *Fusobacterium*, which co-aggregates with different microorganisms in the oral cavity, influencing the state of periodontal health and disease [20].

The presence of *F. nucleatum* in human breast cancer tissue has been established undoubtedly. The role of *F. nucleatum* in promoting breast cancer progression was well studied in cell lines, animal models, and in patients by Parhi et al. and other authors [21,22,23]. Other papers reported that *F. nucleatum* in human BC tissue was more frequently found in malignant samples compared to benign samples, so *F. nucleatum* was considered a risk factor for the development of BC [19,24]. The recent literature suggests different routes through which the bacterium promotes BC progression [25,26]. To understand how *F. nucleatum* colonizes BC, it is essential to recognize that BC creates a tumor microenvironment (TME) favorable for *F. nucleatum* to grow [27]. BC-associated bacteria are reported to reside intracellularly, both in epithelial and immune cells [2,21,28]. The current literature suggests that *F. nucleatum* creates inflammation and immune evasion within the breast tissue microenvironment, transforming it for the first time into an immunosuppressive tumor microenvironment (ITME) [29]. For more than 10 years, *F. nucleatum* has been considered an oncobacterium in colon cancer, due to its ability to activate oncogenetic pathways [30,31,32,33]. In the following years, other researchers have further investigated the ability of the bacterium as an immunomodulator, due to its action in causing immune evasion [26,34,35,36]. In this current review, for the first time, the composition of the microbiome in healthy breast tissue, the milk microbiome, and their interactions with the inflammatory system and estrogens have been examined. Furthermore, the interaction between the reduction of bacterial load in healthy breasts, modulated during breastfeeding, together with the presence of mutations in high-penetrance genes such as BRCA1 and BRCA2, has been considered as increasing the risk of BC. In this landscape, *F. nucleatum* is not only a bacterium that contributes to the development and progression of cancer. In this review, we therefore focused on the dual role of *F. nucleatum* as an oncobacterium and immune modulator in BC, thus defining it as an onco-immune modulator bacterium. We also considered the changes in the breast during the woman’s reproductive life and the most common genetic alterations affecting the breast.

## 2. Microbiota in Breast Feeding in Healthy and Inflammatory Conditions

**Figure 2:**
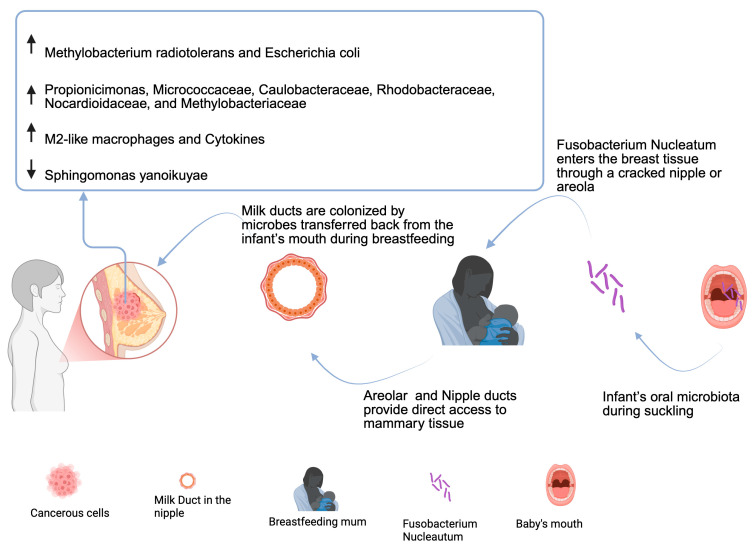
**Retrograde inoculation of *F. nucleatum* into the lactating mammary gland**. This schematic illustrates the retrograde inoculation route, whereby microbes from the infant’s oral cavity are transferred back into the mammary ducts during breastfeeding. This process occurs as negative pressure and peristaltic tongue movements during suckling allow milk and saliva to transiently flow back into the milk ducts. Microbial genera such as *Streptococcus*, *Veillonella*, *Fusobacterium* (species *nucleatum*), and *Actinomyces* have been identified as prominent oral-derived taxa colonizing breast milk via this mechanism [38,41]. This bidirectional exchange contributes to the shaping of both the milk microbiome and the infant gut microbiota, enhancing microbial diversity and potentially supporting immunological development. The figure also shows the changes in the microbiota of a cancerous breast environment. Image created with BioRender (https://biorender.com; last accessed on 18 July 2025).

The breast microbiome plays a specific biological role rather than being a random accumulation of external microbes [37]. It is composed of a diverse array of bacteria, many of which are beneficial for infant health by supporting immune system maturation, metabolic programming, and protection against pathogens [38]. The bacterial communities in breast milk contribute significantly to the establishment and development of the neonatal gut microbiome. Research indicates that breast milk contains a dynamic and evolving microbiota, influenced by maternal factors such as diet, mode of delivery, and health status [8]. Key bacterial genera found in both breast milk and infant feces include *Bifidobacterium* (supports gut health and immune regulation), *Lactobacillus* (promotes gut barrier function), *Streptococcus* (plays a role in early microbial colonization), and *Staphylococcus* (common but varies depending on maternal health) [39] (Figure 2). The human milk microbiota (HMM) also consists of a diverse range of bacterial species, as well as viruses, fungi, archaea, and protozoa that contribute to neonatal gut colonization [39]. Of interest, *Proteobacteria* is the principal phylum in human milk, with many of the same bacteria that we detected in tissue, raising the possibility that the tissue microbiota may contribute to the infant’s early gut colonization and may be a source of bacterial inocula for babies [40].

In a recent cohort study, the presence of *Bifidobacterium breve* in the maternal rectum, breast milk, and infant stool further supports the entero-mammary pathway [42]. Human milk contains not only bacteria but also a complex virome and mycobiome, both of which may influence infant microbial colonization and immune development [43,44]. An increasing number of studies support the existence of a “gut–lung axis”—a bidirectional communication network linking the intestinal and respiratory microbiota—which underscores the role of human milk in modulating immune responses across multiple mucosal sites during early life [42]. This axis reinforces the idea that breast milk not only supports gut microbial colonization but may also influence respiratory immunity and neonatal health outcomes through systemic immunological crosstalk [45].

Notably, microbial species found in maternal feces, breast milk, and the infant gut frequently overlap, suggesting the existence of multiple transmission pathways. These include the entero-mammary route, whereby maternal gut bacteria translocate across the intestinal barrier to the mammary gland via systemic circulation and lymphatic transport [46]; retrograde inoculation from the infant’s oral microbiota during breastfeeding; and the transference of microbes from the maternal skin [47]. The identification of butyrate-producing bacteria such as *Faecalibacterium*, *Roseburia*, and *Coprococcus* spp. in both maternal fecal samples and breast milk further underscores the significance of gut-derived microbial seeding in establishing the infant’s early microbiome [46]. These taxa are known to play a role in the maturation of the gut epithelium and regulation of host immune responses.

The composition of the HMM is significantly influenced by the mode of milk delivery, whether it is expressed and fed via a pump or delivered directly from the breast through breastfeeding. Direct breastfeeding facilitates dynamic microbial exchange between mother and infant, particularly through retrograde milk flow during suckling. This process appears to favor colonization by beneficial bacteria such as *Bifidobacterium*, which are less abundant in pumped milk [48]. In contrast, expressed milk, due to its exposure to external surfaces, storage containers, and variable temperatures, tends to harbor more environmental or opportunistic microbes [49].

Environmental factors also play a role: rural breast milk has been shown to contain greater microbial diversity than urban samples, likely due to broader environmental exposures including diet, hygiene, and contact with nature [50].

Beyond these influences, human milk is enriched with complex carbohydrates known as human milk oligosaccharides (HMOs), which play a central role in supporting a healthy infant microbiome [51]. Although indigestible by the infant, HMOs selectively promote the growth of commensal bacteria, particularly *Bifidobacterium* spp., which, in turn, contribute to gut maturation and immune system development [52]. The interplay between HMOs and milk microbiota fosters mucosal immunity, strengthens epithelial barrier function, and promotes the development of gut-associated lymphoid tissue mechanisms that are fundamental in protecting against infections and immune-related diseases [53,54]. The long-term implications of these interactions are increasingly evident in epidemiological studies. Azad et al. [55], in a comprehensive review, underscored the immunomodulatory potential of human milk, linking its microbial and bioactive components to reduced risks of allergic and autoimmune diseases. Taken together, these findings position the breast microbiome as a central actor in the developmental programming of infant immunity. Through its influence on microbial colonization, immune cell maturation, and the orchestration of diet–microbe–host interactions, the HMM serves not only as a conduit of maternal microbes but also as a dynamic and adaptive system that shapes the health trajectory of the infant well beyond the breastfeeding period.

Breast dysbiosis is strongly associated with mastitis. Research by Jiménez et al. [56] identified an over-representation of pathogenic species such as *Staphylococcus aureus* and *Streptococcus* spp. in women experiencing mastitis, suggesting that an imbalance in the local microbiome can trigger inflammatory cascades and compromise breastfeeding continuity (Figure 1). Beyond infection, alterations in breast microbiota have also been observed in women with obesity. Collado et al. [57] reported that obese mothers exhibit distinct microbial signatures in both colostrum and mature milk, with implications for milk composition, metabolic programming, and the vertical transmission of microbiota to the infant. In neonates, the implications of dysbiosis in early breast milk exposure are profound. Adequate microbial colonization in the first months of life is pivotal for the development of the gut microbiome, which, in turn, influences metabolism, immune regulation, and long-term health trajectories. An inadequate or imbalanced initial microbiota, especially one lacking key taxa such as *Bifidobacterium* or *Lactobacillus*, has been linked to the development of metabolic disorders, childhood obesity, and immune-related conditions including allergies and asthma [58]. Taken together, these findings underscore the importance of preserving the integrity of the breast microbiome through supportive maternal care, informed breastfeeding practices, and judicious use of medications such as antibiotics that may alter microbial balance. Further research into the dynamics of breast microbiota composition and its functional implications will be critical for the development of interventions aimed at optimizing maternal–infant health outcomes.

Women living with autoimmune and chronic inflammatory diseases, such as inflammatory bowel disease (IBD), represent another population where the immunological and microbial composition of breast milk may be altered. Immunosuppressive therapies and systemic inflammation have the potential to modify breast milk’s bioactive profile, though data remain limited. Preliminary studies indicate that despite these alterations, breastfeeding may continue to offer immunological benefits to the infant, including the transmission of protective antibodies and immune-modulating cytokines that may partially counteract the infant’s risk of inflammatory disorders [59].

The psychosocial state of the lactating parent is increasingly recognized as a modulator of the HMM [41]. Disruptions to the hypothalamic–pituitary–adrenal (HPA) axis can impair epithelial barrier integrity within the breast, facilitating shifts in microbial colonization. Reduced microbial diversity and altered immune signaling in the milk of stressed mothers could have downstream effects on infant development, including immune function, stress reactivity, and even gut–brain axis programming [60].

These insights collectively underscore the dynamic interplay between maternal health and the microbial constitution of human milk. Understanding the influence of inflammatory states on HMM not only aids in optimizing breastfeeding support strategies but also contributes to more targeted interventions that safeguard maternal and infant health.

Looking ahead, emerging research is uncovering the complex interplay between the human milk virome and mycobiome, that is, the communities of viruses (including bacteriophages) and fungi (e.g., *Malassezia* and *Candida*) present in breast milk [61,62]. These non-bacterial components contribute not only to the microbial richness of milk but also to its immunomodulatory properties [48]. Bacteriophages, for example, may help regulate bacterial populations in the infant gut, while fungal elements might influence immune responses and barrier function [63]. This broader ecological view challenges the traditionally bacteria-centric model of microbiome science and suggests that human milk serves as a rich, biologically active fluid-delivering not just nutrition, but also a sophisticated toolkit of microbial agents and regulatory factors.

## 3. Molecular Alterations in Breast Cancer

BC has emerged as the most commonly diagnosed female neoplasm in all age groups and the leading cause of cancer death among women worldwide [64] The etiology of BC is multifactorial, with both endogenous and environmental factors potentially interacting in its pathogenesis. Risk factors for BC include genetic mutations, late menopause, obesity, and reproductive factors [65,66,67].

BC is a complex molecular disease involving alterations to several cellular pathways that control cell growth and proliferation. Affected pathways include MAPK, RB/E2F, PI3K/AKT/mTOR, and TP53 [68]. The complex molecular pathways involved in BC progression originate from deregulated crosstalk controlling cell growth and renewal. These pathways include HER2, c-MYC, the estrogen receptor (ER), cyclins D1 and E, TP53, and the phosphatase and tensin homolog (PTEN). They are also affected by the failure to repair DNA damage that occurs during physiological processes, such as those involving the BRCA1 molecular network [69,70].

The presence of hereditary factors significantly increases the likelihood of developing BC. The risk of developing BC due to pathogenic mutations in high-penetrance genes, such as BRCA1 [OMIM 113705] and BRCA2 [OMIM 600185], has been recognized for several decades [71,72]. Some of the genetic risk can be explained by pathogenic variants in other BC susceptibility genes, including TP53, CDH1, PALB2, and PTEN, and various rare gene variants have also been reported to increase the risk of developing BC [73,74]. However, the presence of multiple polygenic susceptibility alleles, resulting in a cumulative risk from low-penetrance mutations, is also a widely recognized risk factor [75,76]. Moreover, several genes related to metabolism, oxidative stress, and inflammation have been linked to an increased risk of BC, including those involved in one-carbon metabolism, estrogen metabolism, and lipid metabolism [77,78,79,80]. Therapeutic strategies have been proposed to benefit patients with mutations in BRCA1/2 and other genes required for homologous recombination (HR). Around 22% of BCs are estimated to exhibit a BRCA1/2 alteration, whereas a functional deficit in the HR system overseen by BRCA1 has been identified in 69% of triple-negative breast cancers (TNBCs). These cancers tend to be more aggressive, carry a higher risk of recurrence, and have a poorer prognosis [81,82]. The estimated lifetime risk of developing BC for carriers of the BRCA1 and BRCA2 mutations is 69% and 72%, respectively. Twenty years after an initial BC diagnosis, the respective risks of developing contralateral BC are 40% and 26% [83]. Estrogens have been associated with an increased risk of BC. However, emerging clinical and experimental evidence suggests that progesterone, whether produced naturally or synthetically, is the primary hormonal factor underlying this risk. In fact, estrogens may indirectly contribute to BC risk by inducing progesterone receptors and amplifying progesterone signaling [84].

In clinical settings, methods based on immunohistochemistry using four surrogate markers, estrogen receptor [ER], progesterone receptor [PR], HER2, and Ki67 are commonly used to identify BC subtypes [85,86]. According to the World Health Organization (WHO) classification system, breast carcinoma is classified as either invasive or non-invasive [87]. Invasive carcinoma accounts for 70–75% of cases and is grouped into four categories based on hormone receptor expression. These categories are ER positive (ER+), PR positive (PR+), human epidermal growth factor receptor positive (HER2+), and TNBC. TNBC is characterized by the absence of expression of any of these receptors. Lobular carcinoma, which accounts for 12–15% of cases, is also characterized by the deregulation of E-cadherin/β-catenin [88]. There are also eighteen other uncommon subtypes, accounting for 0.5–5% of cases. Pathological descriptions include the tumor’s histological type and grade, as well as an immunohistochemical assessment of its hormone receptor status (ER and PR), HER2 expression, and Ki67 expression [89].

BC is a heterogeneous group of diseases that respond differently to various personalized treatment modalities [90,91]. The most common method used in clinical practice is a molecular classification approach using gene expression profiling: luminal A, luminal B, enriched HER-2, and basal-like or TNBC [92]. Because each subtype has unique responses and prognoses to therapeutic interventions, a subtype-specific treatment approach is necessary [92].

The progression of BC is determined by a number of factors, including the characteristics of tumor cells, elements of the TME (both cellular and non-cellular), and the characteristics of the surrounding tissue. Significant advances have been made in the treatment and diagnosis of primary BC in recent years [93]. Alongside traditional imaging techniques and pathological diagnostic methods, liquid biopsies, multiple immunofluorescence tests, and digital pathology approaches are being increasingly adopted in clinical practice. Various treatment options are available for BC, and recent clinical studies emphasize the importance of personalized, targeted therapies [70,94]. The long-term follow-up management of BC patients is also crucial, as this can impact therapeutic outcomes and improve quality of life [95]. Whether or not the tumor has spread to the sentinel lymph nodes (SLNs) is a key factor in predicting the outcome of BC. The SLNs are usually the first place to which the cancer spreads, and patients with lymph node metastases (LNMs) have lower overall and disease-free survival rates, regardless of the type of BC [96,97,98]. The spread of tumor cells to the draining lymph node can lead to lymphangiogenesis and the trafficking of cytokines and chemokines. This can result in immune evasion and alterations to the TME, leading to the expansion of cancer cells [99]. Several studies are currently investigating the factors in tumor and lymph node microenvironments that drive metastatic capacity [100]. Systemic treatments that prevent the development of distant metastases are ineffective for certain types of BC, such as TNBC. Metastatic disease is the leading cause of death for most BC patients. The prolonged latency period between initial treatment and recurrence suggests that tumor cells adapt to and interact with molecular signals from the primary tissue microenvironment and the host systemic environment. This process facilitates disease progression and the invasion of other organs, such as the liver, lung, brain, and bone [101].

Recent studies suggest that exposure to environmental chemicals with endocrine-disrupting properties may increase a woman’s risk of developing BC at certain stages in her life. Significant structural and functional changes occur in the mammary gland during critical periods, including prenatal development, puberty, pregnancy, and menopause. Furthermore, alterations in the mammary microenvironment and hormone signaling can enhance BC susceptibility [102]. Alterations in gut microbial diversity that cause dysbiosis have been linked to BC development. These alterations modulate host immune responses and inflammatory pathways, thereby promoting tumorigenesis and progression. Additionally, differences in gut microbiota populations have been observed between women with and without BC, further implicating the role of gut microbiota in cancer development [103]. Changes in estrogen metabolism, a key factor in BC development, have also been linked to the composition of the gut microbiota [104]. Gut microbiota expressing the enzyme β-glucuronidase (GUS) can increase estrogen bioavailability by deconjugating estrogen–glucuronide moieties [105]. This process enables estrogen to be reabsorbed into the bloodstream. Increased circulating estrogens can induce estrogen receptor positivity in BC cells. Furthermore, GUS-expressing microbiota can affect the effectiveness and toxicity of anticancer therapies by altering glucuronide-conjugated drug metabolites. Consequently, GUS inhibitors have emerged as a potential anticancer treatment [106]. Further studies are needed to determine how carcinogenesis, tumor marker expression, and endocrine receptor-targeted therapies affect the microbiota and vice versa.

## 4. The Microbiota in Breast Cancer

Among all immunity cells, M2-like macrophages are the most present in the BC microenvironment and are related to lower survival in HR + BC patients [82]. These cells have been observed to infiltrate into BC tissue and in healthy closed mammary glands during the initial and late stages of cancer progression. Interestingly, a rich and various microbiome has been detected in BC [77]. There are many analyses that indicated a deep, different microbiome of the breast between cancerous and healthy tissues and between benign and malignant cancers [7,19,21], demonstrating that changing the microbial community can influence BC progression [19].

In recent years, there has been a great interest in the characterization of the microbiota of various areas of the body and under different health conditions; this is because different studies have shown that bacterial communities are different between areas of the body and that complex interactions between host and bacteria are established [107,108].

Xuan et al. [11] detected a different presence of the genera *Methylbacterium* and *Sphingomonas* across paired healthy and/or normal adjacent tissue and cancerous tissue; this may propose that they could have a role in cancer progression. *Sphingomonas yanoikuyae* is present in healthy breast tissue, while it reduces dramatically in tumor tissue, where instead, *Methylobacterium radiotolerans* is the most enriched bacterium in cancerous tissue [109]. In tumor tissue, *Methylbacterium radiotolerans* was relatively abundant and predominant (found in 100% of samples), whereas in paired normal *tissue, Sphingomonas yanoikuyae* was relatively abundant and predominant [11]. The inverse correlation of these two bacteria in normal and tumor breast tissues is evidence that dysbiosis is associated with breast cancer [11].

In women with cancer, there is a higher amount of *Escherichia coli* than in healthy controls, which is known for its ability to promote cancer [110]. A study by Constantini et al. [111] shows that the genus *Ralstonia* is present in breast tissue, including in tumors and adjacent normal tissue. A significant abundance of *Propionicimonas*, *Micrococcaceae*, *Caulobacteraceae*, *Rhodobacteraceae*, *Nocardioidaceae*, and *Methylobacteriaceae* has been highlighted in an Asian cohort of BC patients [109]. Another study underlined a reduction in *Bacteroidaceae*, while an increase in *Agrococcus* has been observed in parallel with the progression of cancer. Additionally, the presence of *Fusobacterium*, *Atopobium*, *Gluconacetobacter*, *Hydrogenophaga*, and *Lactobacillus* genera has been associated with cancer progression [112]. A recent study on a cohort of TNBC patients has showed an important presence of *Clostridiales* in cancerous tissue related to a triggered immune microenvironment [113].

The evidence of a metabolically active tissue-resident microbiota is supported by the presence of bacteria correlated with the production of the metabolite trimethylamine N-oxide (TMAO), capable to stimulate antitumor immunity mediated by CD8+ T cells and M1 macrophages [114].

The gut microbiome involved in several processes: it modulates inflammation and influences cell genomic stability through the dysregulation of different pathways, but it is also linked to the progression of cancer by acting on estrogens metabolism through enterohepatic circulation [115]. The interaction between the human microbiome and cancer is referred to as the “oncobiome”. It has been suggested that some microbes may play a role in breast carcinogenesis by promoting anticancer immunity and immune surveillance and/or modulating systemic estrogens levels [11,116].

The associations between BC and estrogen levels could reflect differences within people’s intestinal microbial communities [117], as published by Adlercreutz and collaborators 50 years ago, who demonstrated the vital role of the gut microbiota [118]. Even more recent is the Fuhrman and co-workers’ study, which shows that postmenopausal estrogen metabolism is associated with microbial diversity [119]. The investigation of the connection between breast cancer and gut microbiota are still ongoing.

The so-called “estrobolome”, comprised of enteric bacterial genes that produce estrogen and its metabolites, was extensively discussed by Plottel and Blaser [120] in 2011. Elevated levels of circulating estrogens and their metabolites can result from perturbations in the microbiota/estrobolome, which can increase the risk of BC. Clinical research has proven that the gut microbiota and urinary estrogens and estrogen metabolites are associated in a positive way [119,121]. Estrogens are metabolized in the liver, where they are conjugated and expelled with the bile into the lumen of gastrointestinal tract; there, they are de-conjugated by bacterial β-glucuronidase, and then they are reabsorbed as free estrogens through enterohepatic circulation, reaching different organs like the breast [115]. These metabolic products are generated through estrogen metabolism made by some bacteria inside the *Clostridia Ruminococcaceae* families [115]. Additionally, there are other metabolites similar to estrogen that are produced by gut oxidative and reductive reaction and by induced synthesis of estrogen-inducible growth factors, which could play a carcinogenic role. Furthermore, bacterial β-glucuronidase may be involved in the deconjugation of xenobiotics and/or xenoestrogens, resulting in their reuptake through the enterohepatic pathway and consequently extending the duration of their stay in the body [122].

The *Firmicutes* phylum contains two dominant subgroups, the *Clostridium leptum* cluster and the *Clostridium coccoides* cluster, where a lot of β-glucuronidase bacteria can be found. The *Escherichia/Shigella* bacterial group, which is a *Proteobacteria* phylum, also has enzymes that metabolize β-glucuronidase [123]. The relationship between gut microbiome and breast cancer risk through estrogen-independent pathways has been investigated in other studies [120,124,125]. Postmenopausal BC patients had less diverse gut bacteria and a significantly altered microbiota composition, as evidenced by a case-control study comparing the fecal microbiota of BC patients with paired controls [125]. The case patients show, in the gut microbiome, higher levels of *Clostridiaceae*, *Faecalibacterium*, and *Ruminococcaceae* but lower of *Dorea* and *Lachnospiraceae*. Furthermore, in cancer patients, the fecal microbiota was less various (α-diversity). Total estrogens are connected to α-diversity only in control patients but not in case patients, and it was surprising to discover that cancer patients had higher, but not statistically significant, levels of systemic estrogens. A possible reason could be found in the other known risk factors for breast cancer like adiposity and obesity, because in these situations, the microbiota appears to be less diversified [126].

It is interesting to note that bacteria like *Listeria fleischmannii* were highly associated with genes responsible for epithelial-to-mesenchymal transition (EMT), while *Haemophilus influenza* was linked to pathways involving tumor growth, cell cycle progression, E2F signaling, and mitotic spindle assembly. These discoveries indicate that the microbiome composition of tumors can be linked to certain intrinsic traits. Despite this, further investigation is necessary for this type of investigation. It is not clear if there is a connection between certain bacteria and mutations that BC cells harbor. The fact that *Escherichia coli*, *Staphylococcus*, and *Bacterioides fragilis* isolated from breast tumors have been described as having clear genotoxic activity is what makes this topic particularly fascinating [7].

In their research, Urbaniak et al. examined breast tissue collected from different parts of the breast in women aged 18 to 90, with or without cancer, and some subjects did not have a history of lactation. The population of bacteria was found to be diverse, but *Proteobacteria* was the main phylum. Viable bacteria were found in certain samples through culture, despite the absence of signs or symptoms among the subjects [12].

From the analogy of normal adjacent tissue of BC woman and tissue of non-cancer women was found a significant increase of *Prevotella*, *Lactococcus*, *Streptococcus*, *Corynebacterium*, and *Micrococcus*, whereas in patients with BC, *Bacillus*, *Staphylococcus*, *Enterobacteriaceae*, *Comamondaceae*, and *Bacteroidetes* were found [127].

These bacteria can cause damage to DNA in vitro. Intestinal bacteria such as *Staphylococcus* and *Enterobacteriaceae* can cause DNA damage in host cells. *Staphylococci* generate the toxin PSMα (phenol-soluble modulin alpha) and specific lipoproteins (Lpls). DNA damage may be caused by PSMα, while Lpls hinder DNA damage repair signaling, which can damage the host cell genome [128]. Like *Staphylococcus*, *Enterobacteriaceae*, particularly those expressing polyketide island synthase (PKS), produce the genotoxin colibactin [129]. Genomic instability in host cells is caused by DNA damage and by p53 inactivation [130].

In addition, a reduction in some lactic acid bacteria was detected, which are well known for their beneficial health results, like anti-carcinogenic qualities [127]. According to these findings, the bacteria or their components may impact the local immune microenvironment, and there may be an unsuspected link between dysbiosis and breast cancer [14].

The Cancer Genome Atlas provided Thompson and co-workers [131] with the information needed to characterize the breast microbiota in 668 breast tumor tissues and 72 non-cancerous adjacent tissues. They also reported potential microbial compositional shifts among the various disease subtypes. *Proteobacteria* was the most prevalent phylum found in tumor sites (48%), while in the vagina, oral cavity, skin, gastrointestinal tract, and bladder, the *Proteobacteria* phylum represents only a small percentage of the global bacterial community, unlike the breast, where it is the most abundant phylum [107,132,133,134]. *Actinobacteria* and *Firmicutes* were less represented, at 26.3% and 16.2%, respectively. The results are coherent with past outcomes from Hieken et al. [19] and Urbaniak et al. [7]. In tumor samples, there was a difference in the abundance of two prevalent species, *Mycobacterium fortuitum* and *Mycobacterium phlei*. The presence of *Proteobacteri*a in tumor tissue samples increased, while *Actinobacteria* was also observed in non-cancerous adjacent tissue samples [131].

A significant number of viruses have been found in breast tumor tissue and/or TME, along with bacteria, parasites, and fungi. Certain authors have suggested that some of these viral signatures could be linked to specific breast cancer subtypes [135].

According to Banerjee and co-workers, an analysis of 100 TNBC samples revealed predominant viral, bacterial, fungal, and parasitic genomic sequence signatures. *Actinomycetaceae*, *Caulobacteriaceae*, *Sphingobacteriaceae*, *Enterobacteriaceae*, *Prevotellaceae*, *Brucellaceae*, *Bacillaceae*, *Peptostreptococcaceae,* and *Flavobacteriaceae* were among the many families linked to cancer [136].

Parhi et al., in an in vitro and in vivo study, definitely demonstrated that among all bacteria, *F. nucleatum* was associated with breast tumor progression [23].

## 5. *Fusobacterium nucleatum* and Its Onco-Immunomodulatory Role in Breast Cancer

*F. nucleatum* is a common oral Gram-negative anaerobe associated with a wide spectrum of human diseases such as periodontal disease [137,138,139], adverse pregnancy outcomes [140,141,142], rheumatoid arthritis [143], and inflammatory bowel disease [144,145]. Its association with cancer was later established. It was first identified in colorectal cancer [30,31,32,33,146] and then was also associated with oral, pancreatic, esophageal, gastric, cervical, and breast cancers [23,147,148,149,150,151,152], hence the term of oncobacterium.

In the colon model, it has been hypothesized that the *F. nucleatum* reaches the organ through the hematogenous route rather than the gastrointestinal route [153]. This transient bacteremia is recurrent in patients with periodontal disease. But it is also likely that oral *Fusobacteria* can enter the circulatory system even during trivial injuries during daily hygiene. At first *F. nucleatum* binds to vascular endothelial cadherin (VE-cadherin) through the surface adhesin A (FadA) that promotes the penetration of *F. nucleatum* into endothelial cells [154,155]. Upon reaching the host cell, *F. nucleatum* binds via its autotransporter protein 2 (Fap2). This protein specifically recognizes Gal-N-acetylgalactosamine (Gal-GalNAc), a sugar abundantly exposed by colorectal cells [153]. It is very likely that *F. nucleatum* may also reach Gal-GalNAc-displaying tumors in other organs via this route. In BC, *F. nucleatum* has been reported to be overabundant, as well as Gal-GalNAc. This suggests a colonization mechanism of *F. nucleatum* like in colon cancer [19,156,157]. *F. nucleatum* enrichment in BC is largely due to Fap2, as demonstrated in Fap2-deficient mutant mice that exhibited a decrease in bacterial colonization compared to Fap2 WT mice [23].

There are three possible routes through which *F. nucleatum* translocates to the mammary gland contributing to carcinogenesis [158]. (1) Due to bleeding gums during periodontal disease, *F. nucleatum,* via transient bacteriemia, penetrates VE-cadherin using its surface FadA, which disrupts tight junctions, increasing permeability. Then, the surface lectin Fap2 recognizes GalNAc overexpressed in tumor cells, and it enters BC cells through the rich blood supply. Furthermore, *F. nucleatum* activates p38, inducing the production of MMPs by tumor cells, which, in turn, activates the invasion, angiogenesis, and metastasis pathways [159]. (2) Regarding the breast–gut axis, oral *F. nucleatum* arrives in the gut through circulation or the lymphatic system. It opens the tight junctions between intestinal epithelial cells, dendritic cells, and CD18+ cells, facilitating their translocation from the intestinal lumen to the breast, especially during pregnancy and lactation [19]. In breast tissue, *F. nucleatum* Fap2 interacts with Gal-GalNAc to enter BC cells. 3) Via direct contact with the nipple/areola during breastfeeding or sexual activity, oral *F. nucleatum* gets access to enter mammary tissue. The nipple–areolar openings represent a potential opportunity for bacteria from the skin and oral cavity to populate the breast tissue during breastfeeding or sexual intercourse [19].

**Figure 3:**
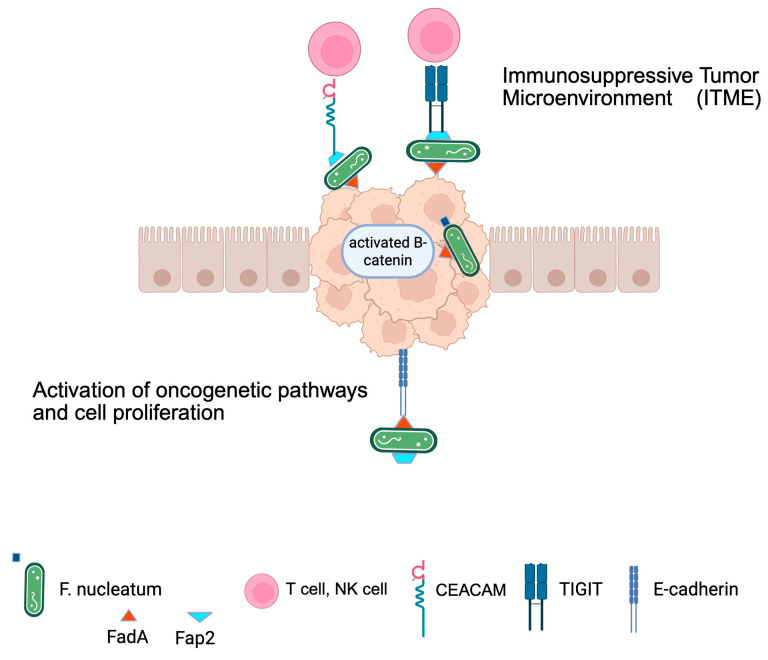
***F. nucleatum* as an onco-immune modulator**. The figure illustrates the dual role of *F. nucleatum.* Protein Fap2 of *F. nucleatum* binds to the cell adhesion molecule CEACAM1 and to immune receptor TIGIT, both expressed by T and NK cells, inhibiting the function of immune cells and causing cancer cells evasion. At the same time, the bacterium, through its adhesin FadA, binds to E-cadherin, activating the Wnt/β-catenin oncogenetic pathway and causing tumor proliferation and progression. Image created with BioRender (https://biorender.com; last accessed on 18 July 2025).

*F. nucleatum* contributes to carcinogenesis by increasing tumor cell proliferation and inhibiting tumor cell apoptosis [26,121,158,160]. In detail, *F. nucleatum* induces a proinflammatory microenvironment that becomes a TME after its binding with E-cadherin (Figure 3). It makes FadA activate the Wnt/β-catenin signaling pathway, thus increasing the transcription of nuclear factor-κβ (NF-κβ) and oncogenes cyclin D1 and c-Myc [30,31]. *F. nucleatum* promotes tumor proliferation not only by activating the Wnt/β-catenin pathway but also by inhibiting apoptosis. *F. nucleatum* also activates β-catenin signaling through the TLR4/PAK1 cascade [161]. The binding of *F. nucleatum* lipopolysaccharide (LPS) to TLR4 activates NF-κβ, which upregulates the expression of genes that inhibit caspase 8. This represses apoptosis [158]. Since TLR4 is highly expressed in BC cells, it is conceivable that *F. nucleatum* may also promote BC progression through a similar, TLR4-dependent mechanism [22].

In addition to contributing to carcinogenesis by activating the oncogenetic pathways described above, *F. nucleatum* modulates immunity against mammary tumors by manipulating antitumor immunity. Several studies have found that *F. nucleatum* mediates the development and immune evasion in BC, although specific connection of the gut–mammary axis in the immune system awaits further investigation. Is it known that in colon cancer cell, Fap2 of *F. nucleatum* binds the human immune receptor named “T-cell immunoreceptor with Ig and ITIM domains” (TIGIT), present on Tumor-Infiltrating Lymphocytes (TILs) [36]. TIGIT regulates T-cell mediated immunity. This binding inhibits T-cell cytotoxic activity on tumor cells, protecting them from immune cell attack (Figure 3).

Another way by which F. nucleatum inhibits T-cell function is through the recruitment of MDSCs [22]. This may be due to increased expression of immune checkpoint inhibitors such as CD47 (cluster of differentiation 47, which inhibits phagocytosis and cytotoxicity of phagocytes on host and cancer cells) and matrix metalloproteinase 9 (MMP-9). Increased infiltration of MDSCs into the tumor environment is associated with cancer progression, as MDSCs have potent immune inhibitory effects. These cells deplete the extracellular environment of amino acids, such as arginine and cysteine, required for T-cell proliferation. MDSCs express PD-L1, which further reduces T-cell activation. Upregulated PD-L1 binds to PD-1 on T cells and inhibits their proliferation.

In addition, MYC upregulation by *F. nucleatum* positively stimulates PD-L1 and CD47 expression in cancer cells [162]. Finally, *F. nucleatum* recruitment of MDSCs into the TME has a dual outcome, leading to immune suppression and tumor progression [22].

Furthermore, *F. nucleatum* also binds a carcinoembryonic antigen-related cell adhesion molecule (CEACAM1) expressed by T and natural killer (NK) cells, thus downregulating the antitumor immune response [36] (Figure 3). This binding is mediated by a trimeric auto-transmitting adhesive called CEACAM-binding protein of *Fusobacterium* (CbpF) [36]. By means of these mechanisms, *F. nucleatum* creates an escaping of tumor cell and thus a TME with immunosuppressive activity, an ITME.

In BC tissues, TIGIT and CEACAM1 expressions are upregulated and downregulated, respectively [163,164]. In early-stage BC, TIGIT was highly co-expressed with other immune checkpoint receptors, like PD-1, cytotoxic T lymphocyte-associated antigen 4 (CTLA-4), LAG-3, and TIM-3. High levels of TIGIT and CTLA-4 are associated with favorable prognosis with longer overall survival and relapse-free survival. Yet, the therapeutic use of antibodies against these receptors has not shown good efficacy but rather high toxicity [138,163].

In BC, *F. nucleatum* promotes the expression of both PD-L1 and CD47 [165]. PD-L1 binds to PD-1 expressed on T cells, thereby inhibiting T-cell proliferation and reducing the secretion of cytokine. Thus, the PD-1/PD-L1 axis is thought to be responsible for cancer immune escape [165,166]. Furthermore, *F. nucleatum* helps BC cells to evade killing by CD8+ T lymphocytes through the NFkb/PD-L1 pathway [26]. In this way, *F. nucleatum* inhibits immune responses and promotes self-tolerance.

Recently, an in vitro and in vivo study removed intratumoral *F. nucleatum* by blocking the immune checkpoint [29]. Furthermore, this study also verified that the intratumoral administration of antibiotics, together with the elimination of *F. nucleatum*, has the potential to reverse the ITME and to prolong the survival of tumor-bearing mice. This therapeutic approach allows for effective elimination of *F. nucleatum* while maintaining eubiosis.

## 6. Conclusions and Future Perspectives

*F. nucleatum* promotes breast carcinogenesis by a dual mechanism. On the one hand, it promotes cell proliferation and inhibits apoptosis. On the other hand, it inhibits the immune response.

In conclusion, *F. nucleatum* might create a breast ITME through activating the human inhibitory receptors TIGIT and CEACAM1, upregulating CD47 and PD-L1 expression, promoting immune tolerance, and recruiting MDSCs for immune migration, immune suppression, and tumor progression.

Considering these results, which demonstrate the presence of *F. nucleatum* in human BC, targeting *F. nucleatum* may hold potential as a therapeutic strategy to enhance cancer treatment.

The experimental therapeutic use of antibodies against TIGIT and CTLA-4 receptors has not given good results, while the use of PD-L1 and CD-47 antagonists might also be more effective in combination with the eradication of *F. nucleatum.* Also, immune checkpoint blockade therapy provided a limited clinical response.

Therefore, further investigation of the biology of *F. nucleatum* and its interactions with TME/ITME and with BC cells harboring mutations is needed.

## Data Availability

No new data were created or analyzed in this study. Data sharing is not applicable to this article.
